# Robotic Shoulder Rehabilitation With the Hybrid Assistive Limb in a Patient With Delayed Recovery After Postoperative C5 Palsy: A Case Report

**DOI:** 10.3389/fneur.2021.676352

**Published:** 2021-09-14

**Authors:** Shigeki Kubota, Hideki Kadone, Yukiyo Shimizu, Hiroshi Takahashi, Masao Koda, Kousei Miura, Hiroki Watanabe, Kenji Suzuki, Yasushi Hada, Yoshiyuki Sankai, Masashi Yamazaki

**Affiliations:** ^1^Department of Orthopaedic Surgery, Faculty of Medicine, University of Tsukuba, Ibaraki, Japan; ^2^Center for Innovating Medicine and Engineering (CIME), University of Tsukuba Hospital, Ibaraki, Japan; ^3^Department of Rehabilitation Medicine, University of Tsukuba Hospital, Ibaraki, Japan; ^4^Department of Neurosurgery, Faculty of Medicine, University of Tsukuba, Ibaraki, Japan; ^5^Faculty of Systems and Information Engineering, University of Tsukuba, Ibaraki, Japan

**Keywords:** postoperative C5 palsy, robotic shoulder rehabilitation, hybrid assistive limb, adjustment effect, erroneous motion pattern

## Abstract

C5 palsy is a serious complication that may occur after cervical spine surgery; however, standard procedures for shoulder rehabilitation for patients with postoperative C5 palsy have not yet been established. We used a wearable robot suit Hybrid Assistive Limb (HAL) in a patient with delayed recovery after postoperative C5 palsy and conducted shoulder abduction training with the HAL. A 62-year-old man presented with weakness in his left deltoid muscle 2 days after cervical spine surgery. He experienced great difficulty in elevating his left arm and was diagnosed with postoperative C5 palsy. Seven months after surgery, shoulder abduction training with a HAL was initiated. In total, 23 sessions of shoulder HAL rehabilitation were conducted until 26 months after surgery. His shoulder abduction angle and power improved at every HAL session, and he was able to fully elevate his arm without any compensatory movement after the 23rd session, suggesting that the HAL is a useful tool for shoulder rehabilitation in patients with postoperative C5 palsy. We employed shoulder HAL training for a patient with delayed recovery from postoperative C5 palsy and achieved complete restoration of shoulder function. We believe that the HAL-based training corrected the erroneous motion pattern of his paralyzed shoulder and promoted errorless motor learning for recovery. Our collective experience suggests that shoulder HAL training could be an effective therapeutic tool for patients with postoperative C5 palsy.

## Introduction

C5 palsy is a severe complication that may occur after cervical spine surgery, with a sudden occurrence of motor loss in the C5 region, resulting in difficulty in elevating the arm (1–6). Fortunately, more than 60% of patients with postoperative C5 palsy achieve complete recovery from motor loss (2, 7); however, in some patients, recovery from C5 palsy is incomplete, and the shoulder dysfunction persists (7, 8). Physical and occupational therapies are common approaches to shoulder rehabilitation post-C5 palsy after cervical spine surgery. Active and passive range of motion (ROM) exercises, muscle strength training for paralyzed and residual muscles, and pain management to prevent shoulder joint contraction and muscle atrophy are the most common treatment methods (9–12); however, the effects of these exercises on recovery from muscle weakness are limited. Standard procedures for shoulder rehabilitation in patients with postoperative C5 palsy have not yet been established. Thus, there is an urgent need to develop a novel rehabilitation technique for C5 palsy to promote early recovery of shoulder dysfunction.

The Hybrid Assistive Limb (HAL) is a wearable exoskeleton robot developed at our institute (13). In addition to the conventional components of a robot, the HAL possesses a specific bioelectrical signal sensor that enables it to support voluntary exercise. Several types of HALs, such as the bilateral leg HAL, unilateral leg HAL, single-joint HAL for the elbow and knee, and lumbar HAL, have been reported in the literature (13).

We previously employed a single-joint HAL in a patient with motor loss associated with the biceps muscle after surgery of the cervical spine; using the HAL, the patient could perform elbow flexion exercises (14). The single-joint HAL was subsequently modified to develop a shoulder rehabilitation system (15). The new system contains sensors that allow the HAL to detect muscle action potentials from the deltoid of the user and assists voluntary shoulder abduction exercises.

We have previously reported the use of the shoulder HAL in a patient with motor loss of one upper extremity caused by a traumatic incomplete cervical spinal cord injury (16). Shoulder rehabilitation with a HAL facilitated early recovery from shoulder abduction dysfunction. In this study, we reported the use of shoulder HAL training in a patient in a critical condition with delayed recovery from postoperative C5 palsy, in whom shoulder dysfunction continued for 7 months after surgery.

## Materials and Methods

### Patient Presentation

**Figure 1:**
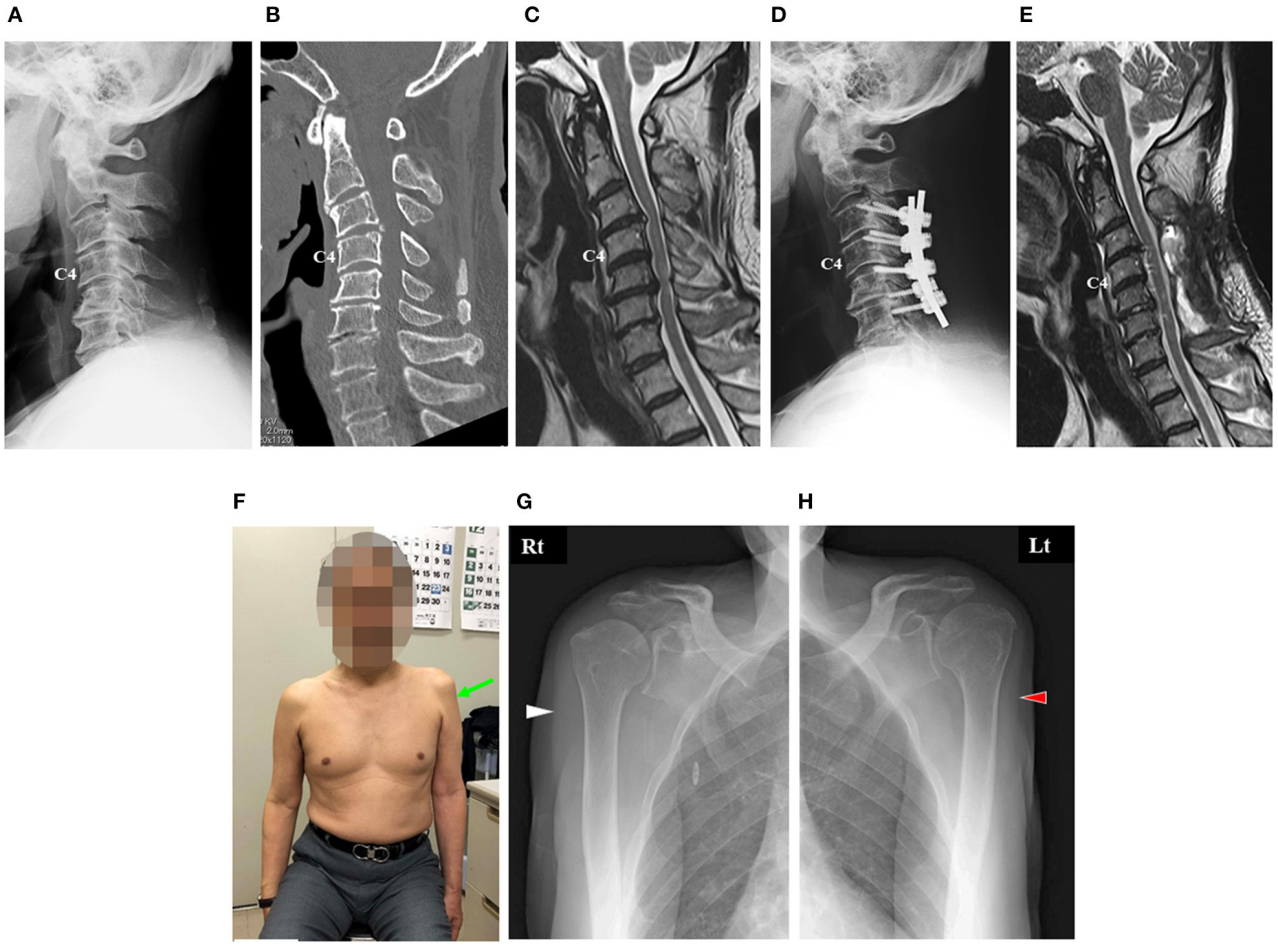
Imaging findings of the cervical spine of the patient before surgery **(A–C)**. Image findings of his cervical spine after surgery **(D,E)**. Photograph and radiographs of the shoulders of the patient 7 months after surgery **(F–H)**. **(A)** Cervical lateral radiograph revealing slight kyphosis of his cervical spine. **(B)** Sagittal reconstruction computed tomography (CT) image revealing disc space narrowing with a posterior spur at C5–C6 and C6–C7 and postdiscal ossification at C3–C4. **(C)** Midsagittal T2-weighted magnetic resonance (MR) image revealing that the spinal cord was severely compressed anteriorly at C3–C4 and C5–C6. **(D)** Cervical lateral radiograph 2 weeks after surgery revealing instrumented fusion at C3–C6. **(E)** Midsagittal T2-weighted MR image 4 weeks after surgery revealing adequate decompression of the spinal cord. **(F)** Anterior view showing muscle atrophy around his left shoulder. **(G,H)** Anterior–posterior radiographs of his right shoulder **(B)** and left shoulder **(C)** showing marked muscle atrophy in his left deltoid (red arrowhead) compared with that in his right deltoid (white arrowhead).

A 62-year-old man underwent posterior decompression with instrumented fusion at C3–C7 for cervical compression myelopathy at another hospital (Figures 1A–C).

The patient complained of neck pain, dexterity in both hands, gait disturbance, and frequent urge to urinate before the surgery. Deep tendon reflexes were hyperactive in both upper and lower extremities, and he showed numbness in both hands. A slight decrease in muscle power in his left arm and leg was observed; manual muscle testing (MMT) of his left deltoid showed a score of grade 5.

A plain lateral radiograph and a sagittal reconstruction image acquired using computed tomography (CT) before surgery showed slight kyphosis of the cervical spine (Figures 1A,B). Similarly, the images showed disc space narrowing with a posterior spur at C5–C6 and C6–C7, as well as postdiscal ossification at C3–C4 (Figures 1A,B). Midsagittal T2-weighted magnetic resonance imaging (MRI) showed severe anterior compression of the spinal cord at C3–C4 and C5–C6 (Figure 1C).

The surgery comprised posterior decompression at C3–C7 and spinal fusion with lateral mass screws at C3–C6 (Figure 1D). At the rod–screw connection, a slight correction of his cervical kyphosis was performed. The surgery was completed without complications. During surgery, spinal cord monitoring was performed, and transcranial electrical motor-evoked potentials showed no abnormalities in the upper and lower extremities.

After surgery, the preoperative symptoms of myelopathy of the patient were relieved. Postoperative MRI showed sufficient decompression of the spinal cord (Figure 1E).

### Development of Postoperative C5 Palsy

The patient experienced pain in his left shoulder a day after the surgery; however, no motor loss was observed. Two days after the surgery, he developed weakness in the bilateral deltoid muscles (MMT grade 2) and biceps (MMT grade 4), suggesting postoperative C5 palsy. He could not elevate his arms independently.

Due to no signs of recovery from C5 palsy 25 days after the first surgery, he underwent additional bilateral foraminotomy at C4–C5 and C5–C6. While he regained the ability to elevate his right arm 3 months after the first surgery, his left arm remained immovable. Nerve conduction and needle electromyography (EMG) studies were performed 3 weeks after the second surgery (6 weeks after the onset of C5 palsy). The left axillary nerve stimulation–left deltoid recording showed that the left deltoid muscle compound muscle action potential was severely reduced. Needle EMG examination showed active neurogenic changes in the left biceps; therefore, radiculopathy in the C5 region with axonal degeneration was strongly suspected. Although he continuously underwent conventional shoulder rehabilitation after the onset of C5 palsy under the supervision of physical and occupational therapists in the hospital, severe left deltoid muscle weakness continued for 7 months after surgery; thus, he was referred to our hospital for shoulder rehabilitation using a HAL.

On his first visit to our hospital, muscle atrophy of the left deltoid was evident (Figures 1F–H). Neurological examination revealed a motor weakness of the left deltoid muscle (MMT grade 2) and biceps muscle (MMT grade 3). We initiated left shoulder abduction training with a shoulder HAL 7 months postoperatively.

### Shoulder Abduction Training With the HAL

**Figure 2:**
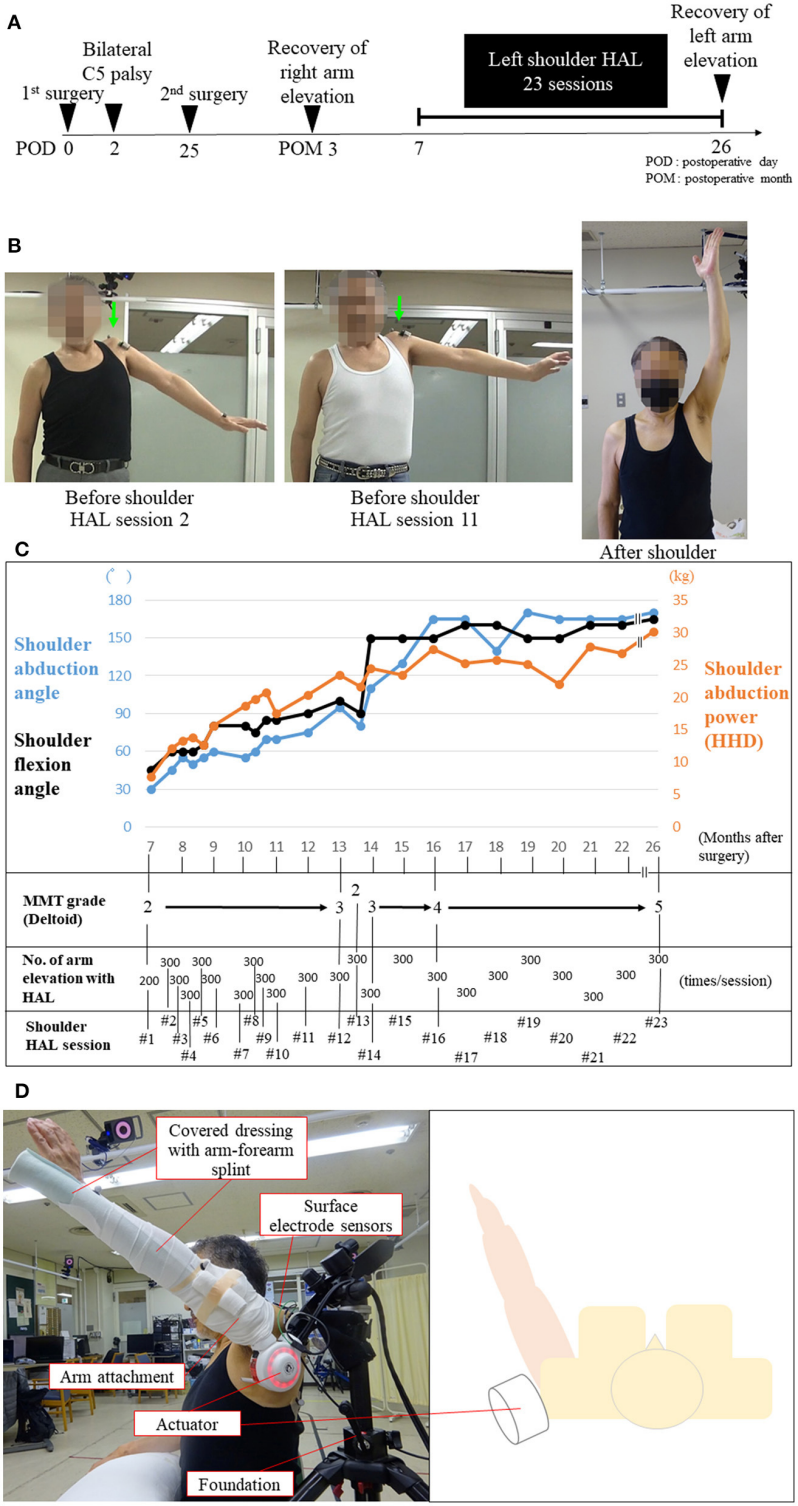
Images demonstrating C5 palsy after surgery and during the shoulder Hybrid Assistive Limb (HAL) training **(A,B)**. Entire process of the shoulder HAL training of the patient **(C)**. Photographs illustrating shoulder HAL abduction training in the scapular plane **(D)**. **(A)** Schematic showing the time course of the development of and recovery from C5 palsy. **(B)** Photograph showing maximum abduction of the left shoulder. Before the second and 11th HAL training sessions, his left shoulder abduction angle was less than 90° while standing, and the deltoid muscle power was classified as manual muscle testing (MMT) grade 2. The arrow indicates the elevation of his left shoulder (shrugging), indicating compensatory movement due to contraction of the trapezius muscle. After the 23rd HAL training session, full abduction of his left shoulder was possible, and the deltoid muscle power improved to MMT grade 5. **(C)** The graph shows the chronological improvement in shoulder abduction and flexion angle and shoulder abduction power using handheld dynamometer (HHD) testing during shoulder HAL training sessions. After HAL training session 14, his deltoid muscle power improved to MMT grade 3 or higher. **(D)** The shoulder HAL consists of 1) an actuator for the shoulder joint on the lateral side, 2) arm attachments, 3) covered dressing with an arm–forearm splint, 4) surface electrode sensors on the deltoid, and 5) foundation.

Between 7 and 26 months after surgery, the patient underwent left shoulder abduction training using a shoulder HAL for 23 sessions, with one session conducted principally once or twice a month, as an outpatient (Figures 2A,C). Additionally, he underwent conventional rehabilitation five times a week [from immediately to postoperative month (POM) 7], three times a week (from POM 8 to 11), and once a week (from POM 12 to 20) in his previous hospital. The conventional rehabilitation continued in his previous hospital from immediately after surgery to POM 20.

The shoulder HAL system comprises an actuator, an arm attachment, a surface electrode sensor, an arm–forearm splint, a manual controller, a battery, and a control device (Figure 2D and Supplementary Video). The shoulder HAL is a wearable exoskeleton-type robot that can be used for voluntary-assisted training in a paralyzed shoulder using an actuator on the lateral side of the shoulder joint and muscle action potentials (bioelectrical signals) detected from the middle fibers of the deltoid muscle. Before shoulder HAL training, his shoulder abduction angle was 30° in the standing position (Figure 2B and Supplementary Video) and 90° in the supine position. The power of left shoulder abduction (deltoid) before shoulder HAL training was classified as MMT grade 2. Before shoulder HAL training, grade 3–4/10 numbness was observed in both hands (C7 and 8 areas). The training method was similar to that reported by Makihara et al. (15). First, the therapists set up the shoulder HAL foundation on the floor, and the shoulder HAL was attached and fixed to the foundation. The arm attachment of the HAL was connected to the shoulder HAL, while the patient sat on a chair placed near the shoulder HAL. Subsequently, an arm–forearm straight splint was fixed on the upper limb of the patient (arm–forearm) in the sitting position. The straight splint, the arm–forearm of the patient, and the arm attachment of the HAL were covered in dressing to avoid a loose attachment between the upper limb of the patient and the HAL. With the patient in the sitting position, the surface electrode sensors of the shoulder HAL were attached to the middle fibers of the deltoid muscle responsible for shoulder abduction. The code of the surface electrode sensors was connected to the control device, and subsequently, the therapists turned on the shoulder HAL. The attachment of the shoulder HAL required approximately 3–5 min to be completed with the assistance of two persons. The therapists regulated the movement and assistive level of the HAL using a manual controller during the shoulder exercises. The shoulder HAL assisted the shoulder abduction motion of the patient according to the timing of the voluntary muscle activity (deltoid muscle) of the patient. The shoulder abduction speed with the use of the shoulder HAL was determined based on the timing and repeated frequency of the voluntary shoulder abductions of the patient. After the shoulder HAL was attached to the left shoulder in the sitting position, shoulder abduction training was initiated in the scapular planes. Subsequently, each training session lasted for 30–45 min and included 200–300 repetitions of shoulder abduction exercises using the shoulder HAL while the patient was in a seated position (Figure 2D and Supplementary Video). The number of repetitions per session was determined according to patient factors, such as fatigue, motivation, and shoulder pain. The level of assistance provided by the shoulder HAL was adjusted by the therapist. First, the therapist adjusted the level of assistance provided by the HAL to achieve the full upper arm elevation of the patient. The level of assistance of the HAL was reduced by the therapist when it became too strong for the patient; in addition, when the patient got tired and the upper arm elevation reduced, the therapist immediately increased the assistance level of the HAL. The HAL settings used for this patient are as follows: the standard mode, assist gain, 50–80; BES balance flexion, 100%; extension, 10%; torque, 70%; and angle range, 0–120°.

### Assessment of Shoulder Abduction Angle and Power

Each training session was carefully monitored to observe any adverse events. The maximum shoulder abduction angle was measured before every shoulder HAL training session, and the left deltoid power was evaluated using a handheld dynamometer (HHD: μ Tas F-1, ANIMA Inc., Tokyo, Japan), as described previously (17). The same examiner (SK) performed these measurements in all the HAL training sessions.

### Assessment of Shoulder Muscle Activity

The muscle activities of the left shoulder were measured in each HAL training session using the Trigo™ Lab wireless surface EMG system (Delsys Inc., Boston, MA, USA). We chose the trapezius (descending part) and deltoid muscles (clavicular part) for evaluation. The EMG activities of these muscles during shoulder abduction were measured immediately before (without HAL) and during training (with the HAL). Their values were band-pass filtered (30–400 Hz), rectified, normalized by maximum activation in each session, integrated by a local time window of 50 ms, segmented into cycles of repeated shoulder abduction motion, cycle-time normalized to 0–100%, and averaged to obtain an average pattern of activation per cycle (referred to as normalized iEMG hereafter) with and without HAL. The mean activity was computed for each trapezius and deltoid muscle with and without the use of the HAL. To evaluate the immediate change in average activity using HAL, the ratio of the difference was computed for each muscle using the following equation:


$$\begin{array}{l} \left(1-\;\frac{EMG_{with}}{EMG_{without}}\right)\times 100 \end{array}$$

where EMG_with_ and EMG_without_ represent the average activity with and without HAL, respectively. To evaluate the immediate change in the balance of activity between the measured muscles, the adjustment rate was calculated using the following equation:


$$\begin{array}{l} \frac{EMG_{with,deltoid}/EMG_{without,deltoid}}{EMG_{with,trapezius}/EMG_{without,trapezius}} \end{array}$$

where “with” and “without” indicate the activity with and without HAL, respectively, whereas “deltoid” and “trapezius” indicate the average activity of the deltoid and trapezius muscles, respectively. Regression analysis was performed between the ratio and the number of sessions to assess the change in the ratio through the course of the HAL training sessions. The data were processed using custom scripts in MATLAB 8.4 R2014b (MathWorks, Natick, MA, USA).

### Assessment of Shoulder Motion

The scapular motion during shoulder abduction was evaluated according to the elevation of the acromion before training (without HAL) in each session. A reflexive marker of a three-dimensional motion capture system (Vicon MX with 16 T20s cameras) was attached to the acromion of the patient. The vertical component of the movement of the marker was segmented into cycles; the highest and lowest positions were detected within each cycle, and the vertical ROM of the acromion was computed as the difference between the highest and lowest positions of the marker. Subsequently, the vertical ROM was averaged among the cycles.

### Statistical Analysis

Paired *t*-tests and regression analyses were performed to verify the statistical significance of the findings. *p* < 0.05 were considered statistically significant, and statistical analysis was performed using custom scripts in R, version 3.6.0 (R Foundation for Statistical Computing, Vienna, Austria).

## Results

### Improvement of Shoulder Abduction Angle and Power

Before the first HAL training session, the patient could not elevate his left arm. When he tried to elevate his arm, we observed a shrugging motion of his left shoulder, which was an unwanted compensatory movement (Figure 2B arrow, and Supplementary Video). At the first session, although the HAL enabled full abduction of his left shoulder, the elevating motion was not smooth (Supplementary Video). During the first and second HAL training sessions, although he felt a sense of incongruity in his shoulder during arm elevation, it was not severe enough to cancel the HAL training. After the third HAL training session, he did not feel any unusual sensations during HAL training. No other severe adverse events directly attributable to shoulder HAL use occurred during training. After the third HAL training session, his arm elevation motion assisted by the HAL became smoother, and more elevation training was possible (Supplementary Video).

After the first HAL training, shoulder abduction power, abduction angle, and flexion angle were markedly increased (Figure 2C). His left shoulder abduction angle and abduction power continued to improve during the HAL training sessions (Figure 2C). The increase in shoulder abduction power measured using HHD was relatively smooth; however, the shoulder abduction angle could not readily reach 90°. Even at the 11th HAL training session, his left increased shoulder abduction angle was <90°, and shoulder shrugging motion could still be observed (Figure 2B). At the 12th HAL training session, his left shoulder abduction angle increased to 90°, indicating an improvement in the muscle power of his left deltoid from MMT grade 2 to grade 3 (Figure 2C).

The MMT grade of his left deltoid was 4 at the 16th HAL training session and 5 at the 23rd HAL training session (Figure 2C). Similarly, the MMT grade of his left biceps was 5 at the 23rd HAL training session. The patient was able to fully elevate his left arm without any compensatory movement after the 15th HAL training session, showing complete recovery of his left shoulder function (Figure 2B, Supplementary Video). After the 23rd HAL training session, improvement (from 3–4 to 5/10) in numbness of both hands (C7 and 8 areas) was observed. Left biceps tendon reflex remained absent after the second surgery and shoulder HAL training. Regarding the impairment of the left upper extremity-related activities of daily living due to left postoperative C5 palsy, improvement in functional independent measure (FIM) motor score (e.g., self-care, dressing upper body) was observed after shoulder HAL training (from 88 to 91/91 FIM motor full score).

### Muscle Activities of Deltoid and Trapezius

**Figure 3:**
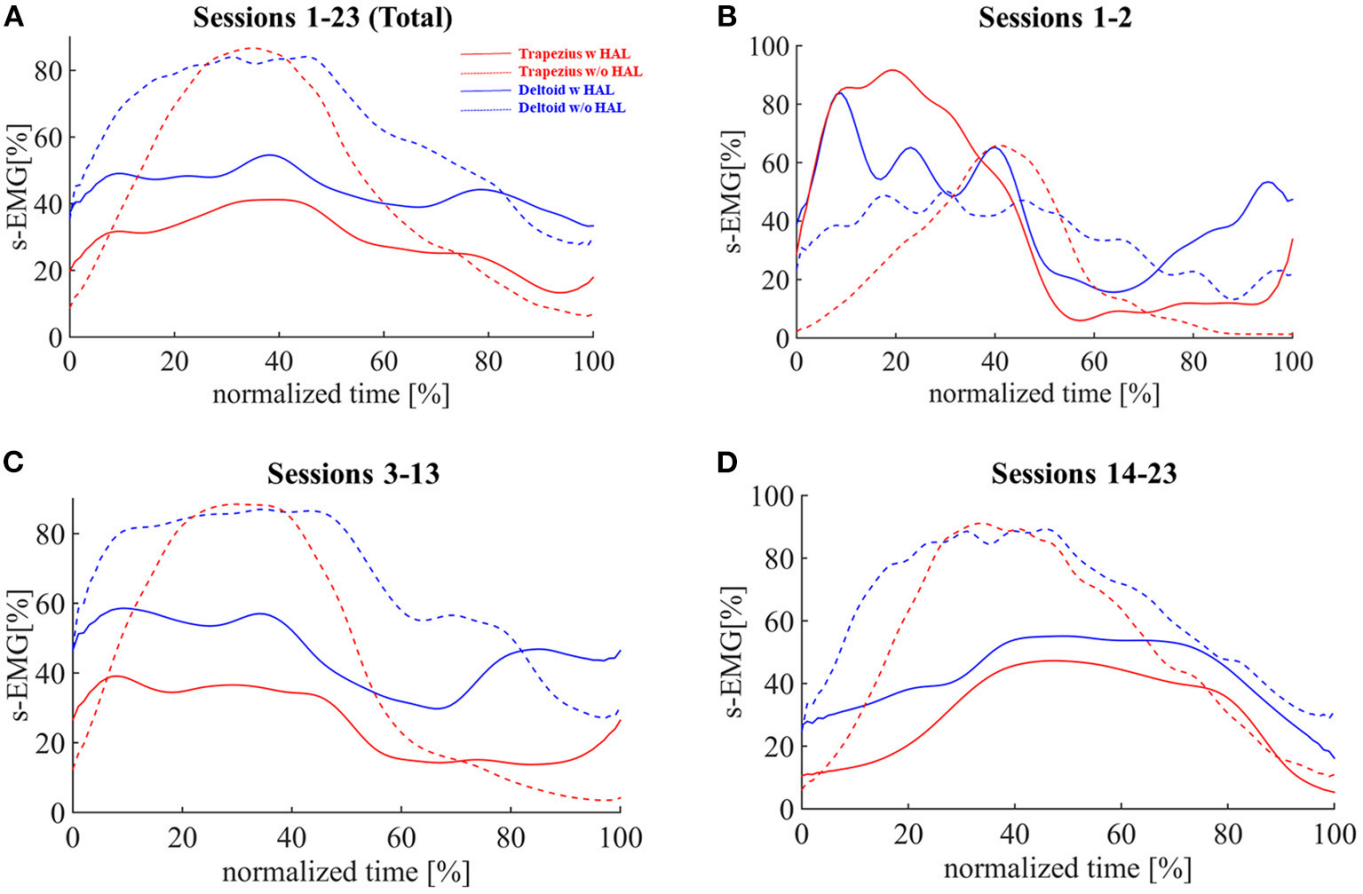
Activities of deltoid and trapezius muscles compared during shoulder abduction training with or without shoulder HAL. Integrated electromyography (iEMG) profiles during the shoulder abduction process. **(A)** Averaged data from HAL training sessions 1–23 (total). **(B)** Averaged data from HAL training sessions 1–2. **(C)** Averaged data from HAL training sessions 3–13. **(D)** Averaged data from HAL training sessions 14–23.

On integrated analyses of shoulder HAL training data averaged through sessions 1–23, the mean muscle activity of the trapezius during arm elevation with the shoulder HAL was significantly decreased by 34.7% (*p* = 0.000013) compared with that obtained with arm elevation without the use of the shoulder HAL (Figure 3A); conversely, the mean muscle activity of the deltoid using the HAL was observed to be slightly lower (decrease by 28.7%, *p* = 0.000403) than that without the use of the HAL (Figure 3A).

Based on the observation in *Improvement of shoulder abduction angle and power*, the analysis was divided into three parts: sessions 1–2, when the patient was initially feeling incongruency during HAL training; sessions 3–13, when he did not experience such feelings and the MMT grade of his deltoid was 2; and sessions 14–23, when the MMT grade was 3 or 4. In sessions 1–2, the mean activity of the trapezius increased by 66.1% and that of the deltoid increased by 27.7% with the use of the HAL (Figure 3B). In sessions 3–13, the mean activity of the trapezius decreased by 39.0% (*p* = 0.00062) and that of the deltoid decreased by 29.5% (*p* = 0.000083; Figure 3C). In sessions 14–23, the mean activity of the trapezius decreased by 40.3% (*p* = 0.000000) and that of the deltoid decreased by 33.0% (*p* = 0.000002; Figure 3D).

**Figure 4:**
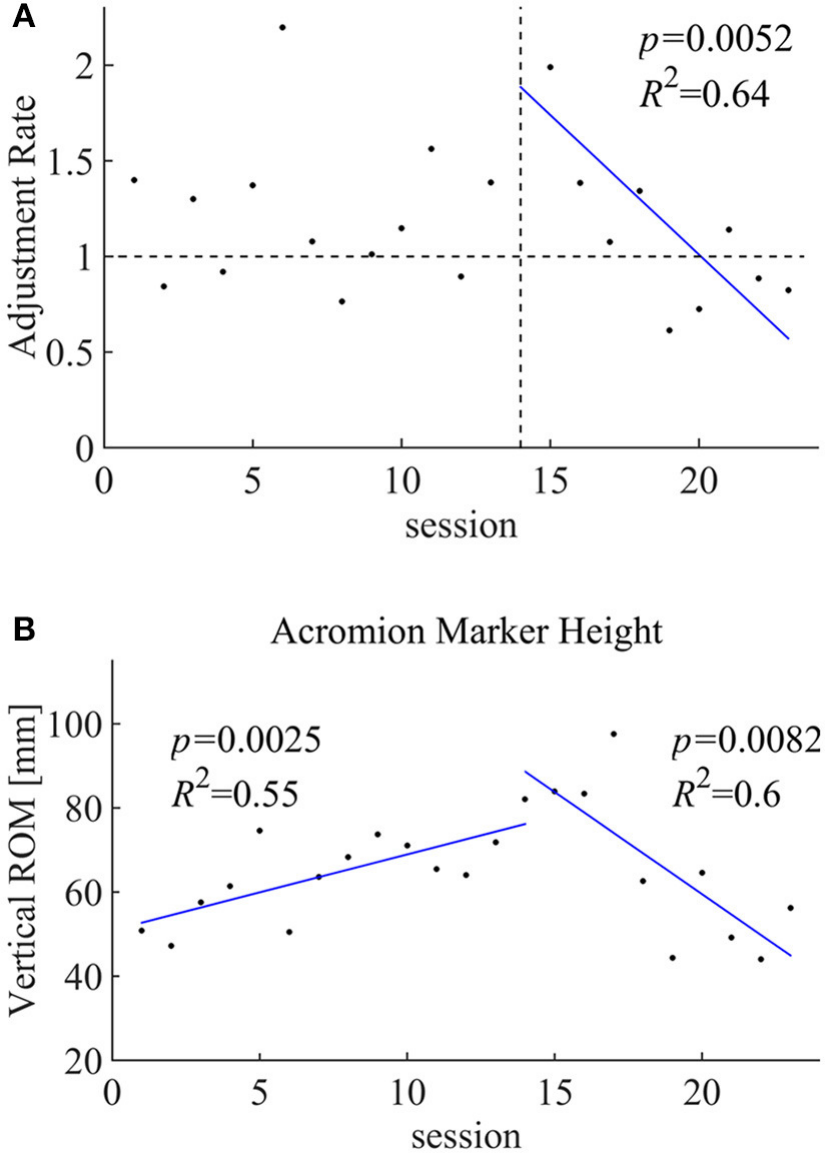
**(A)** Correlation between the adjustment rate and the shoulder HAL training sessions. **(B)** Acromion marker height during the shoulder HAL training sessions.

The adjustment rate was greater than one on average from sessions 1 to 23 (average rate 1.22, *p* = 0.02646). Regression analysis of the rate against the session numbers did not show statistical significance when all sessions were considered; however, for sessions 14–23, we observed a negative correlation (regression coefficient −0.146, *p* = 0.0052, *R*^2^ = 0.64; Figure 4A).

### Shoulder Motion

The vertical ROM of the acromion was evaluated during shoulder abduction without the HAL (Figure 4B) in each session. Regression analysis of the session numbers of the segmented sessions showed a positive correlation (regression coefficient 1.80, *p* = 0.0025, *R*^2^ = 0.55) for the earlier sessions (1–13) and a negative correlation (regression coefficient −4.86, *p* = 0.0082, *R*^2^ = 6) for the later sessions (14–23).

## Discussion

In the present case, after the onset of postoperative C5 palsy, the patient underwent additional foraminotomy and continued to undergo conventional rehabilitation therapy. Despite the patient being treated using extensive therapies for postoperative C5 palsy, shoulder dysfunction persisted for a long period. At 7 months after surgery, the muscle power of the deltoid was classified as MMT grade 2, and the deltoid muscle was markedly atrophic. In such a condition of delayed recovery from C5 palsy, we applied shoulder abduction training using a HAL. With the assistance of the HAL, he was able to elevate his arm and perform voluntary shoulder abduction exercises. After HAL training, the muscle power of the deltoid and shoulder abduction angles gradually increased. Although it took 16 months after the start of HAL training, his shoulder function recovered completely.

With our findings, a question arises: Did the recovery from C5 palsy in this patient occur spontaneously or was it induced by shoulder HAL training? Many studies have described the prognosis of postoperative C5 palsy (18, 19). According to their reports, more than 60% of patients with C5 palsy spontaneously attain complete recovery of shoulder function (18, 19); however, recovery is incomplete in 20–30% of patients (19), and in some patients, no recovery is observed (7). Regarding the factors related to poor recovery, Lim et al. indicated that a deltoid muscle power classified as MMT grade ≤ 2 at onset, the presence of multisegment paresis involving more than the C5 root, and loss of somatic sensation with pain are factors that influence the duration of recovery from postoperative C5 palsy (18). Additionally, a meta-analysis by Sakaura et al. reported that patients with postoperative C5 palsy who are severely paralyzed (MMT grade ≤ 2) required a significantly longer recovery time than that required by patients who are mildly paralyzed (MMT grade = 3 or 4) (18).

Imagama et al. analyzed 1,858 cervical laminoplasty cases and found that 43 patients (2.3%) developed severe postoperative C5 palsy of the deltoid muscle power MMT grade <2 (7). Among the 43 patients, 29 (67%) achieved complete recovery, and the time to recovery from the onset was 3 days to 17 months (mean: 4.1 months). However, they did not reveal chronological changes in the deltoid muscle power in the complete recovery cases. Therefore, based on their data, we are uncertain when the deltoid muscle power of a patient is MMT grade <2 at 7 months after the onset of C5 palsy and the probability of the patient attaining complete recovery after that.

We previously analyzed 199 patients who underwent cervical anterior decompression and fusion surgeries and found that 10 patients (5.9%) developed severe C5 palsy with deltoid muscle power classified as MMT grade <2 at the onset (20). Among the 10 patients, five patients recovered completely within 3 months, two patients recovered to MMT grade 4 within 6 months, and one patient recovered to MMT grade 3 at 6 months and then to MMT grade 4 at 15 months. The remaining two patients did not recover; their deltoid muscle power persistently remained at the level of MMT grade 2 at 6 months after onset. This finding indicated that when the deltoid muscle power was MMT grade ≤ 2 at 6 months after the onset of C5 palsy, it is likely that no patient will improve to an MMT grade >3 thereafter.

In the present case, the deltoid muscle power of the patient was MMT grade 2, and morphologically, his deltoid muscle was markedly atrophic at 7 months after surgery. Considering the findings from previous reports, we believe that the possibility of spontaneous recovery of shoulder function would have been quite low at the time point immediately before the start of HAL training. If we had not applied the HAL at that point, we think that apparent recovery may not have occurred, and severe shoulder dysfunction would have persisted.

In the present case, we evaluated the shoulder function of the patient by measuring the muscle activities of the deltoid muscles. Before HAL training session 1, when he tried to elevate his left arm, the muscle activity of his deltoid was extremely low. This indicated that his deltoid muscle was severely paralyzed and hardly responded to the intention of the patient of shoulder abduction. We suggest that a prolonged period of C5 paralysis caused disuse atrophy of the deltoid. After the first application of the HAL, the patient was able to voluntarily elevate his arm with the assistance of the HAL despite the low muscle activity of the deltoid. During the first HAL training, it was noteworthy that the muscle activity of the deltoid was 4.2 times higher than that before HAL training (Figure 3B). We speculate that voluntary shoulder abduction training assisted by the HAL activated his disused deltoid muscle and oriented it to restore its original function.

Throughout the following sessions, the activities of both the deltoid and trapezius muscles were reduced on average (*Muscle activities of deltoid and trapezius*, Figures 3A,C,D), while the rate of reduction was greater in the trapezius than in the deltoid muscles, as depicted in the adjustment rate greater than one on average (Figure 4A). We believe that this contributed to the patient learning muscular utilization of shoulder abduction while reducing unnecessary activation (Figure 2B).

The deltoid muscle of the patient accomplished normal shoulder abduction motion for the first time in the last 7 months with assistance from the HAL. We suggest that such a successful experience of shoulder abduction in the present patient triggered the progress of his subsequent shoulder abduction exercises. With the assistance of the HAL, the patient was able to continue errorless motor learning and finally attained complete recovery of shoulder function. Thus, we believe that complete recovery of shoulder function in the present patient was not spontaneous, but was achieved due to functional regeneration therapy using HAL.

In HAL training sessions 1 and 2, the trapezius muscle activity increased during the HAL training compared to without HAL. This is a characteristic finding, being different from that observed at the shoulder HAL training in the acute phase of C5 palsy. We previously performed the shoulder HAL training for a patient with C5 palsy derived from acute spinal cord injury (21). In that patient, the muscular activity of the trapezius was preferentially suppressed during the shoulder HAL training in all HAL training sessions, including the first and second HAL training sessions. Based on these data, we suggest that the condition of the shoulder muscles in patients with delayed recovery from C5 palsy is not necessarily similar to that in patients with acute phase C5 palsy. Actually, the present patient described that he felt a certain sense of incongruity during the HAL training in the first and second HAL training sessions. In patients with delayed recovery from C5 palsy, an inappropriate image of erroneous shoulder motion pattern may seem to be deeply impressed not only in their shoulders but also in their brains.

In the present case, the patient was able to achieve complete recovery of shoulder function. However, we believe that the recovery process may not have been satisfactory. After starting HAL training, the patient required 7 months to achieve 90° shoulder abduction and 19 months to recover full power of his deltoid (MMT grade = 5). We consider that if the HAL training was started at an earlier stage after the onset of C5 palsy, his shoulder function could have been recovered much earlier.

Most patients with postoperative C5 palsy undoubtedly achieve complete spontaneous recovery. Thus, it is not practical to apply the shoulder HAL training to all patients with postoperative C5 palsy. Instead, HAL training should be initiated for patients with C5 palsy in whom spontaneous recovery is not expected.

Many studies have so far analyzed postoperative C5 palsy. However, the prognosis of C5 palsy is not yet fully understood, making it difficult to provide the patient with accurate predictions regarding the possibility of complete recovery and the time required for recovery.

More data should be collected from patients with postoperative C5 palsy, and their prognosis should be extensively analyzed. Such studies will enable us to determine the appropriate indications for shoulder HAL training for postoperative C5 palsy. Simultaneously, further research is needed on shoulder HAL training for patients with various stages of C5 palsy to clarify the appropriate timing of initiating shoulder HAL training.

## Study Limitations

This study is a single case report and does not aim to determine the effect of the shoulder HAL training on patients with C5 palsy. In other words, this study was not a controlled trial and could not compare the efficacy of the shoulder HAL training with conventional rehabilitation or spontaneous recovery from C5 palsy. To evaluate the specific effect of the shoulder HAL training, the study should be repeated, recruiting more patients with delayed recovery after postoperative C5 palsy.

## Conclusion

We applied shoulder HAL training for a patient with delayed recovery from postoperative C5 palsy and achieved complete restoration of shoulder function. We believe that HAL training corrected the erroneous motion pattern of his paralyzed shoulder and enabled errorless motor learning for recovery. Our experience suggests that shoulder HAL training could be an effective therapeutic tool for patients with postoperative C5 palsy. A study involving more patients will be needed to evaluate the isolated effects of shoulder HAL training. Moreover, further studies are similarly needed to compare the effectiveness of shoulder HAL training and conventional rehabilitation in the management of C5 palsy.

## Data Availability Statement

The original contributions presented in the study are included in the article/Supplementary Material, further inquiries can be directed to the corresponding author/s.

## Ethics Statement

The studies involving human participants were reviewed and approved by The ethics committee of our university (TCRB18-38). The patients/participants provided their written informed consent to participate in this study. Written informed consent was obtained from the individual(s) for the publication of any potentially identifiable images or data included in this article.

## Author Contributions

SK and HK collected, analyzed, and interpreted the data. SK, HK, KM, and MY wrote and drafted the manuscript. SK also administered HAL therapy and collected clinical scores. MY also organized the study. YuS and HW supported HAL therapy. HT, MK, and YH provided important comments on the planning and implementation of HAL treatment. KS provided essential insights for the analysis. YoS originally developed the robot suit HAL and conceived the idea of HAL therapy. All authors made critical revisions of the manuscript and approved the final version.

## Funding

This work was supported by the Industrial Disease Clinical Research Grants of the Ministry of Health, Labour and Welfare (grant no. 14060101-01). This work was also supported by Grants-in-Aid for Scientific Research of the Japan Society for the Promotion of Science, grant no. 20K19303.

## Conflict of Interest

YoS is the CEO and stockholder of Cyberdyne Inc. The remaining authors declare that the research was conducted in the absence of any commercial or financial relationships that could be construed as a potential conflict of interest.

## Publisher's Note

All claims expressed in this article are solely those of the authors and do not necessarily represent those of their affiliated organizations, or those of the publisher, the editors and the reviewers. Any product that may be evaluated in this article, or claim that may be made by its manufacturer, is not guaranteed or endorsed by the publisher.

## Supplementary Material

Supplementary Video Abduction of the patient's left shoulder: 1) before shoulder HAL training, 2) during shoulder HAL training (sessions 1, 2, 7, and 15), and 3) after shoulder HAL training session 23.

## Abbreviations

CT: computed tomography
EMG: electromyography
HAL: Hybrid Assistive Limb
HHD: handheld dynamometer
MMT: manual muscle testing
MR: magnetic resonance
MRI: magnetic resonance imaging
ROM: range of motion.
